# A Meta‐Analysis on the Efficacy of Noninvasive Positive Pressure Ventilation Combined With Pressure Support Ventilation in Treating Chronic Heart Failure

**DOI:** 10.1002/clc.70041

**Published:** 2025-01-16

**Authors:** Xiaohong Zhang, Ye Dong, Dongliang Diao, Ming Li

**Affiliations:** ^1^ General Practice Chengde Central Hospital Chengde China; ^2^ Research Office Chengde Central Hospital Chengde China

**Keywords:** chronic heart failure, clinical efficacy, meta‐analysis, noninvasive ventilation, pressure support ventilation

## Abstract

**Objective:**

To evaluate the clinical efficacy and safety of noninvasive positive pressure ventilation combined with pressure support ventilation (NPPV‐PSV) in the treatment of chronic heart failure (CHF) through a meta‐analysis.

**Methods:**

A systematic search was conducted using PubMed, Embase, Web of Science, Cochrane Library, CNKI and Wanfang databases to find randomized controlled trials and cohort studies on NPPV‐PSV treatment for CHF. The period of search was set from inception until 2024. Eligible studies were included in a systematic review and meta‐analysis.

**Results:**

A total of 8 studies with 568 patients were included in this meta‐analysis. The meta‐analysis revealed that compared with conventional treatment, NPPV‐PSV treatment had significant advantages in several aspects: clinical efficacy rate (total effect *Z* = 5.10, OR = 3.12, 95% confidence interval (*CI*) [2.01, 4.83], *p* = 0.000), heart rate (HR) (total effect *Z* = 16.26, MD = −10.50, 95% *CI* [−11.76, −9.23], *p* = 0.000), respiratory rate (RR) (total effect *Z* = 16.50, MD = −6.44, 95% CI [−7.20, −5.67], *p* = 0.000) and oxygen saturation (total effect *Z* = 12.40, MD = 0.09, 95% *CI* [0.08, 0.11], *p* = 0.000).

**Conclusion:**

Noninvasive positive pressure ventilation combined with PSV treatment significantly improves clinical symptoms, reduces HR and RR and increases oxygen saturation in patients with CHF, showing superior effects compared with conventional treatment.

## Graphical abstract

A total of eight studies with 325 patients with heart failure were included in this meta‐analysis to investigate the efficacy of noninvasive ventilation combined with pressure support. The results showed that the combination had superior effects in symptoms improvement compared to conventional treatment.

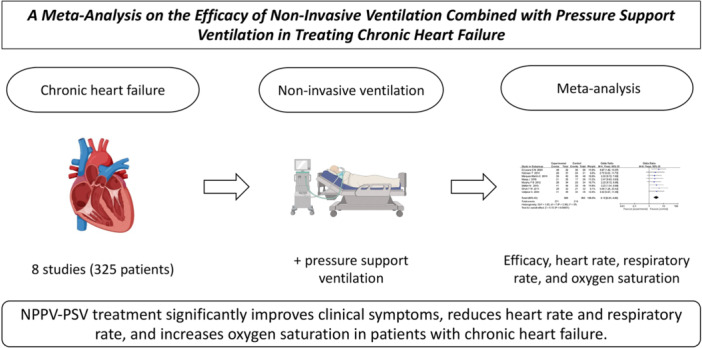

## 1 Introduction

Chronic heart failure (CHF) is a syndrome caused by structural or functional abnormalities of the heart. It is characterized by the heart's inability to pump blood effectively to meet the body's metabolic needs, leading to a series of symptoms and signs, including dyspnea, fatigue, and fluid retention [1]. This condition is a major public health problem in the field of cardiovascular diseases globally, with high incidence, hospitalization, and mortality rates [2]. With an aging population, the incidence of CHF is increasing annually, imposing a heavy economic burden on society and families. While pharmacological treatment is fundamental in the management of CHF, as the disease progresses, medication alone often fails to adequately control the symptoms and improve prognosis [3]. Consequently, the application of nonpharmacological therapies in CHF treatment has garnered increasing attention. Recently, noninvasive positive pressure ventilation (NPPV) combined with pressure support ventilation (PSV) has emerged as a significant adjunctive therapy, recognized for its ability to improve respiratory function, reduce cardiac load, and enhance the quality of life [4]. NPPV, administered through nasal or facial masks, maintains appropriate airway pressure to assist breathing. Its application can effectively alleviate dyspnea, improve oxygenation, and reduce carbon dioxide retention, thereby easing the cardiac burden [5]. Pressure support ventilation aids breathing by adjusting the pressure difference between inhalation and exhalation [6]. The combination of NPPV and PSV leverages the advantages of both modalities, providing sufficient ventilatory support without increasing the patient's respiratory effort, thus improving both respiratory and cardiac functions. Despite the promising potential of NPPV‐PSV in CHF treatment, current studies on its efficacy yield inconsistent results [7]. Some studies report significant improvements in symptoms, quality of life, hospitalization periods, and mortality rates. However, others fail to demonstrate notable benefits, even suggesting potential adverse effects, such as airway dryness and mask discomfort [8, 9]. Differences in patient selection, treatment protocols, and follow‐up durations across studies further contribute to result heterogeneity. Therefore, a systematic review and meta‐analysis are urgently needed to comprehensively evaluate the efficacy and safety of NPPV‐PSV in treating CHF, providing scientific evidence for clinical decision‐making. This study aims to perform a systematic review and meta‐analysis of existing studies to evaluate the efficacy and safety of NPPV‐PSV in the treatment of CHF, including randomized controlled trials (RCTs) and cohort studies published domestically and internationally, with a quantitative synthesis of their results.

## 2 Research Methods

### 2.1 Literature Search Strategy

The search timeframe was from database inception until 2024, including both English and Chinese databases: PubMed, Embase, Web of Science, Cochrane Library, CNKI and Wanfang. The specific search strategy was as follows: (Noninvasive Ventilation) OR (NIV) OR NPPV AND (Pressure Support Ventilation) OR (PSV) AND (Chronic Heart Failure) OR (CHF) OR (Heart Failure, Chronic) OR (Cardiac Failure, Chronic) OR (Chronic Cardiac Failure).

### 2.2 Inclusion and Exclusion Criteria

The inclusion criteria were as follows: patients aged 18 years or older and diagnosed with CHF according to the 2022 guidelines of the American Heart Association and the Heart Failure Society of America. Inclusion required a confirmed diagnosis of CHF with a stable condition, no acute exacerbation or other severe complications and treatment with NPPV‐PSV. Follow‐up was required to last at least 6 months, with regular evaluation of treatment effects.

The exclusion criteria included the following: (1) acute diseases, such as acute heart failure or other acute cardiovascular events (e.g., myocardial infarction or acute myocarditis), (2) severe comorbidities, such as severe renal or liver insufficiency or malignant tumors, (3) respiratory system diseases, such as chronic obstructive pulmonary disease or pulmonary fibrosis, which might affect the evaluation of NPPV‐PSV efficacy, (4) mental diseases, such as uncontrolled severe depression or schizophrenia, which might affect compliance and treatment evaluation, (5) allergy history, specifically severe allergies to Noninvasive ventilators or related treatment equipment, (6) pregnant or breastfeeding women, due to potential adverse effects on the fetus or infant, and (7) any other situations deemed inappropriate for inclusion by the researchers, such as poor compliance or inability to complete follow‐up.

#### 2.2.1 Exclusion Criteria for Literature

The exclusion criteria for the literature included the following: conference abstracts, case reports, reviews, non‐Chinese or non‐English articles, and duplicate publications; studies that did not provide original research data or whose original data could not be obtained; and studies involving patients with consciousness disorders, severe intellectual disabilities, severe anxiety, life‐threatening hypoxemia, the need for immediate intubation or severe obstructive airway diseases.

#### 2.2.2 Intervention Measures

The control group received conventional CHF treatment, including high‐flow oxygen, vasodilators, diuretics, analgesics and electrolyte balance maintenance. The treatment group, in addition to conventional treatment, received noninvasive mechanical ventilation using the BiPAP Vision ventilator in bilevel positive airway pressure (BiPAP) mode.

#### 2.2.3 Outcome Measures

The observed efficacy indicators included respiratory rate (RR), heart rate (HR) and changes in blood oxygen levels before and after treatment. Treatment was considered effective if symptoms such as orthopnea, severe dyspnea, cyanosis, severe cough, pulmonary rales, and wheezing disappeared or significantly improved, with HR, RR, and arterial blood gas analysis returning to near‐normal levels. Treatment was considered ineffective if the above criteria were not met, requiring invasive mechanical ventilation or continued observation and treatment.

### 2.3 Literature Quality Assessment

Two researchers independently evaluated the quality of the included literature, comparing and discussing their results. If a consensus could not be reached, a third researcher participated in the discussion to make a final decision. The quality evaluation was based on the *Cochrane Handbook for Systematic Reviews of Interventions*, considering the following criteria: whether the randomization method was appropriate, whether allocation concealment was implemented, whether blinding was used, the reporting of losses and dropouts along with their reasons, whether an intention‐to‐treat analysis was performed and baseline comparability. Literature quality was graded into three levels: A (low risk of bias, meeting all criteria), B (moderate risk of bias, meeting some criteria), and C (high risk of bias, meeting few or none of the criteria).

### 2.4 Statistical Methods

Meta‐analysis was performed using the analysis module in RevMan 5.3 (Cochrane Collaboration, Copenhagen, Denmark). Relative risks (RRs) were analyzed with a 95% confidence interval (CI). The statistical heterogeneity of the included studies was assessed using *I*² statistics and chi‐squared tests. Heterogeneity was considered significant if *I*² > 50% or *p* < 0.10. When significant heterogeneity was present, a random‐effects model was used to calculate the overall RRs or standardized mean difference with a 95% CI; otherwise, a fixed‐effects model was applied. The possibility of publication bias was evaluated by visual inspection of funnel plots. Outcome measures were continuous variables and were expressed as mean differences or weighted mean differences, with 95% CIs.

## 3 Results

### 3.1 Search Results

**Figure 1:**
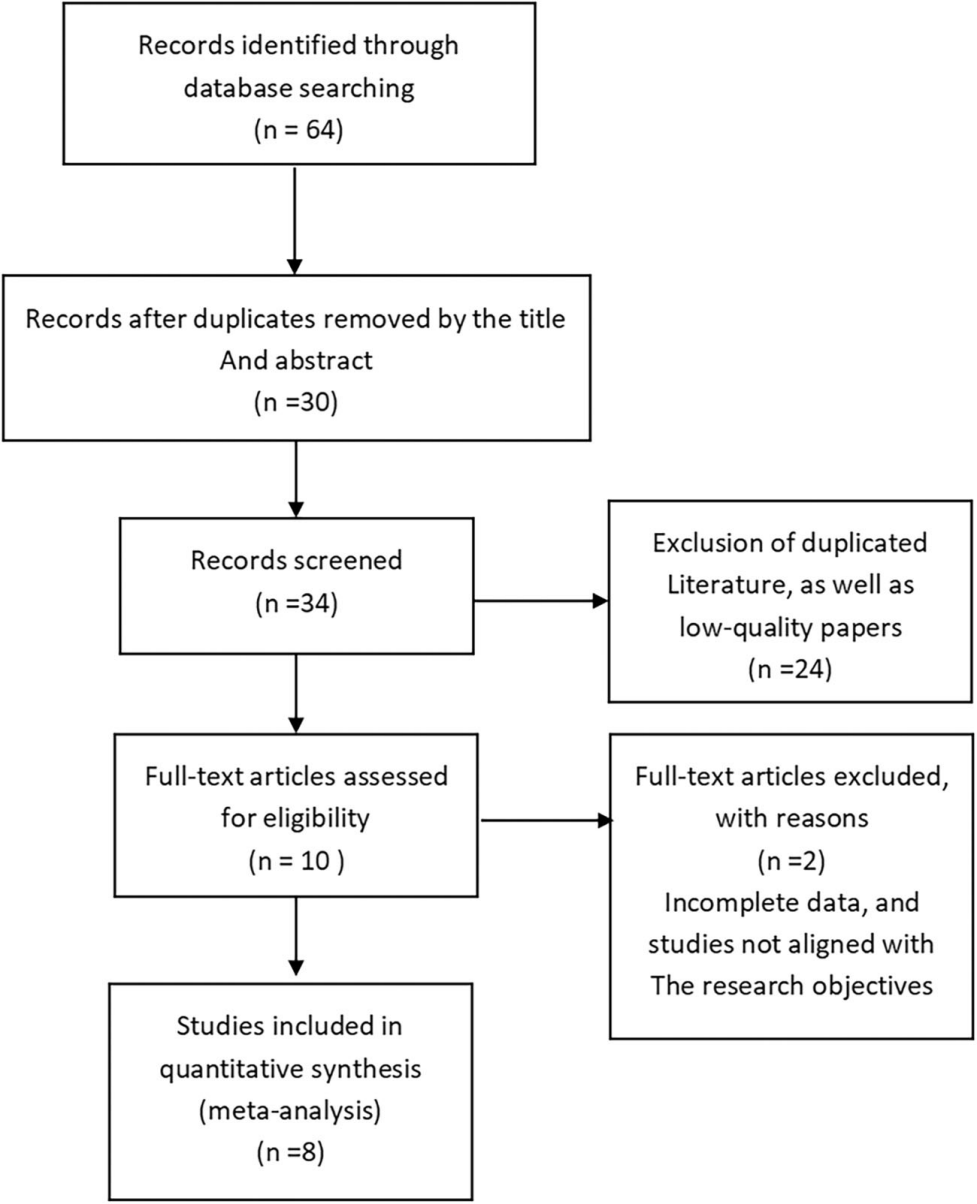
Flow diagram of literature screening process.

A total of 64 articles were initially retrieved. After a thorough review, 10 RCTs met the inclusion criteria. Two duplicate RCTs were excluded, resulting in eight studies being included in the final analysis. Among these, four RCTs described the randomization methods in detail, whereas the other four did not specify their randomization methods. Three RCTs implemented blinding, and all RCTs provided information on whether the allocation concealment was performed. One RCT did not report the reasons for dropouts or losses, making it a low‐quality study. The supplementary table contains detailed information (Figure 1).

### 3.2 Quality Assessment of the Included Studies

**Figure 2:**
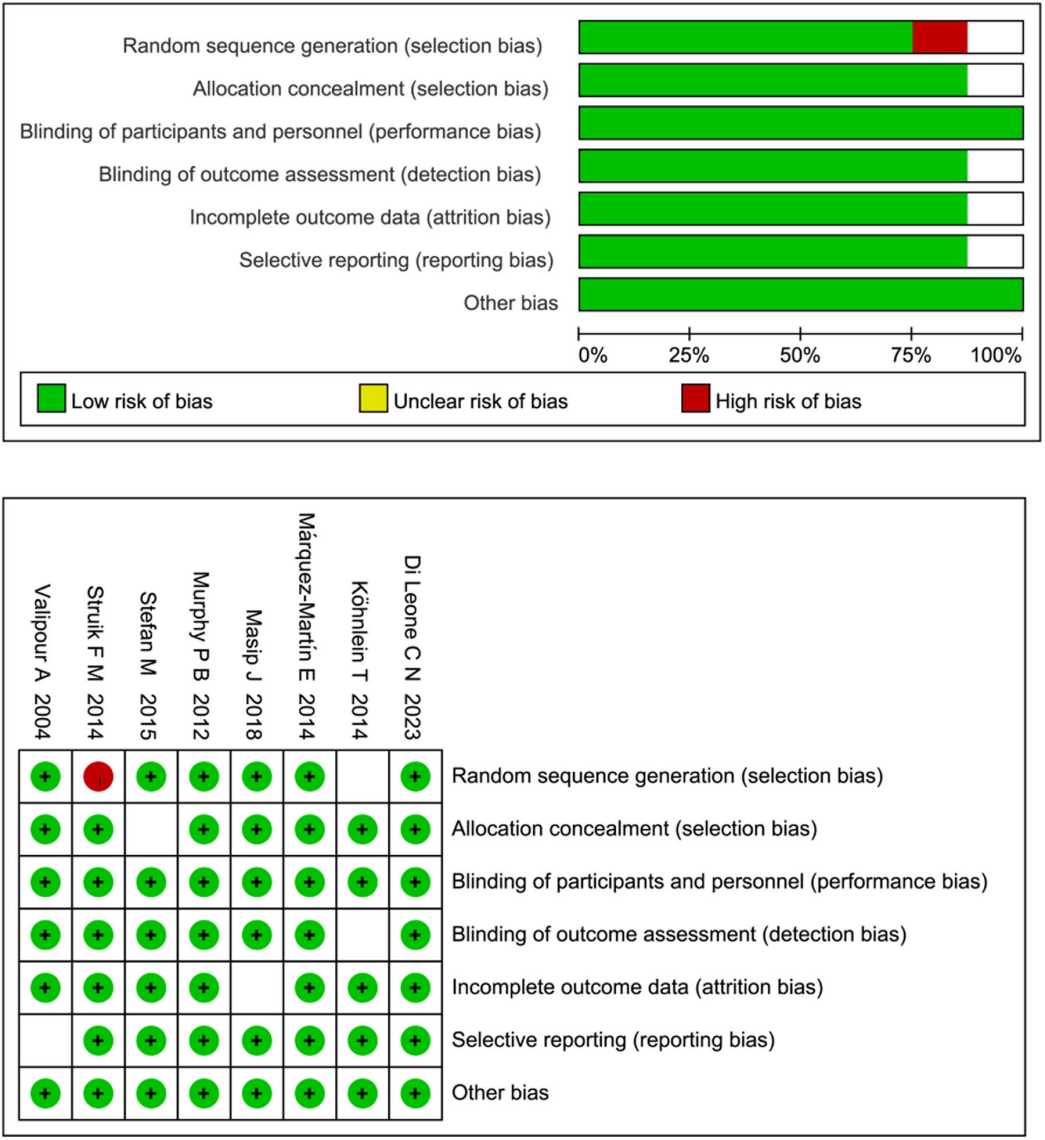
Quality assessment of included studies.

Of the eight included studies [10, 11, 12, 13, 14, 15, 16, 17], four were graded as high‐quality (Grade A) based on methodological rigor, three were of moderate quality (Grade B) and one was of low quality (Grade C). Three studies provided detailed descriptions of their specific methods, and two studies reported on the methods used to conceal allocations. All eight studies had comparable outcome measures and were classified as RCTs (Figure 2).

### 3.3 Characteristics of the Included Studies

The meta‐analysis included eight RCTs. The control groups received standard CHF treatment, including high‐flow oxygen, vasodilators, diuretics, analgesics, and electrolyte balance maintenance. In the intervention groups, in addition to standard treatment, the participants received noninvasive mechanical ventilation using the BiPAP Vision ventilator in BiPAP mode. The duration of BiPAP Vision ventilator intervention ranged from 43 to 60 min (Table 1).

**Table 1:** characteristics of included studies.

References	Year	Location	Sample size	Age	Outcome	Intervention duration, min	Value of reference
Valipour A	2004	America	45/45	≧ 28	①②	54	A
Masip J	2018	Netherlands	25/25	≧ 18	①②③	60	A
Murphy P B	2012	America	34/34	≧ 42	①②	43	C
Struik F M	2014	America	32/32	≧ 35	①②③	45	B
Stefan M S	2015	America	32/32	≧ 30	①②	55	B
Köhnlein T	2014	France	31/31	≧ 40	①③	58	A
Márquez‐Martín E	2014	Germany	40/40	18–60	①②③	60	B
Di Leone C N	2023	United Kingdom	45/45	≧ 50	①②③	55	A

*Note:* ①Clinical Efficacy Rate; ②Respiratory Rate; ③Heart Rate (HR); ④Changes in Blood Oxygen Levels.

### 3.4 Clinical Efficacy Rate

**Figure 3:**
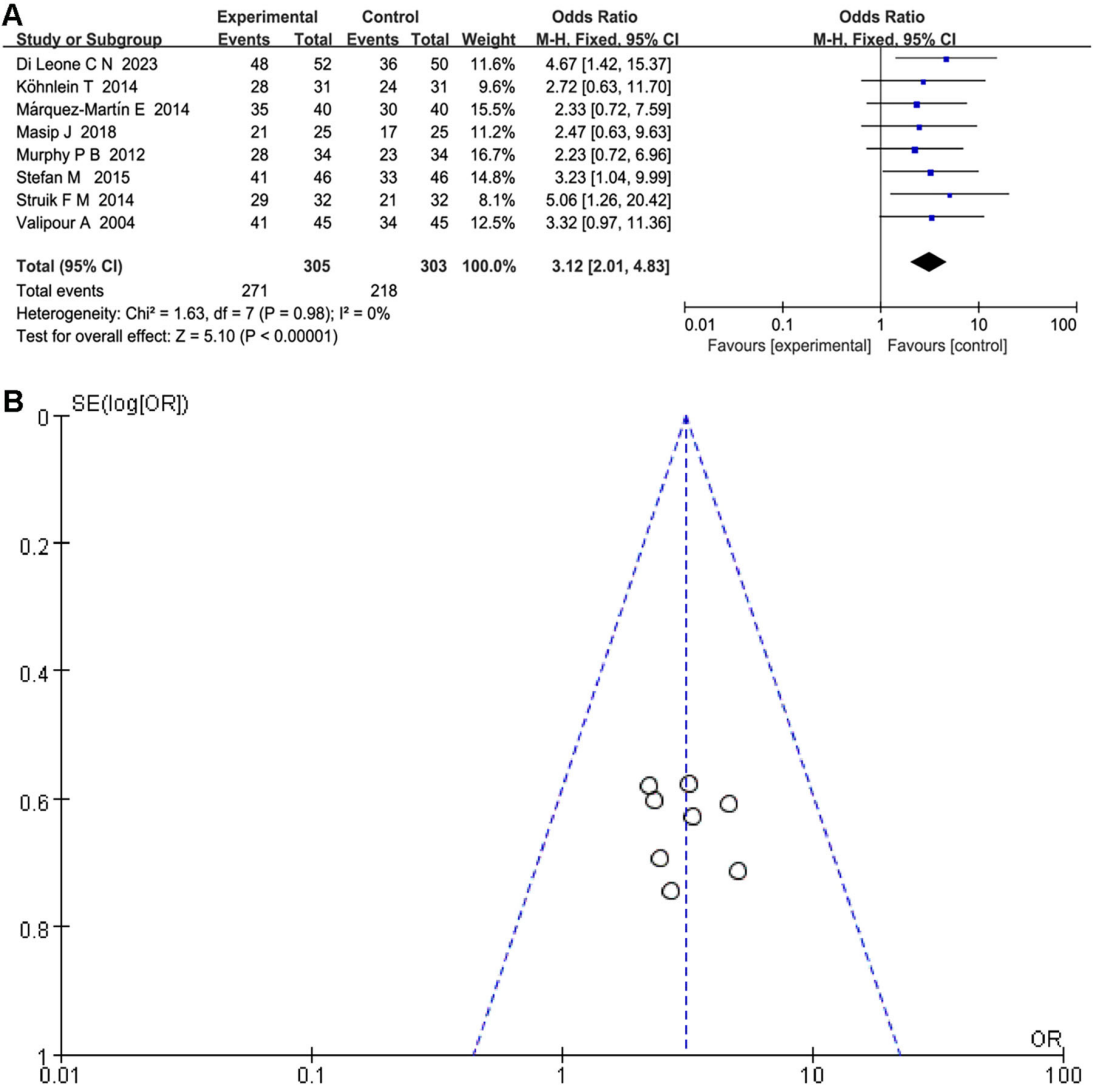
(A) Forest plot of clinical efficacy rate using fixed‐effect model analysis. (B) Funnel plot of the meta‐analysis.

A fixed‐effect model analysis was conducted (*p* = 0.98, *I* ^2^ = 0%) to determine whether NPPV‐PSV improved the clinical efficacy rate of CHF patients in eight studies involving 608 patients. The forest plot results showed that, in the NPPV‐PSV treatment group, 271 out of 305 patients experienced effective treatment, whereas in the conventional treatment group, 218 out of 303 patients experienced effective treatment. The difference between the two groups was statistically significant (total effect *Z* = 5.10, OR = 3.12, 95% *CI* [2.01, 4.83], *p* = 0.000), indicating that NPPV‐PSV significantly improved the clinical efficacy rate in patients with CHF compared with conventional treatment (see Figure 3A). The funnel plots showed no significant asymmetry for all included studies, indicating that the results of the meta‐analysis were stable (Figure 3B). NPPV combined with PSV treatment can significantly improve the clinical efficacy of patients with CHF, improve their prognosis, and provide a better choice for treatment.

### 3.5 Heart Rate

**Figure 4:**
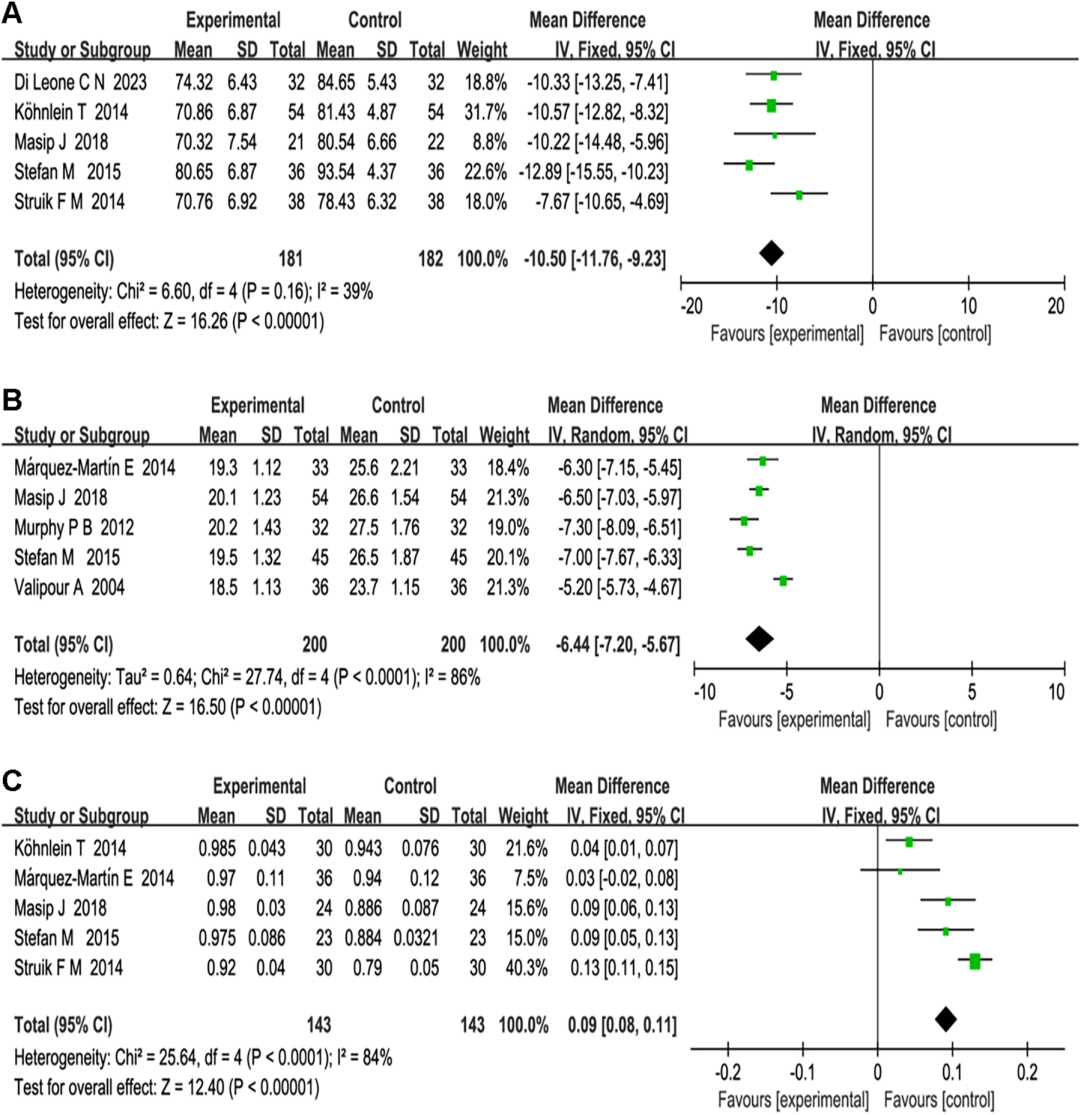
(A) Forest plot of heart rate using fixed‐effect model analysis. (B) Plot of respiratory rate using random‐effect model analysis. (C) Forest plot of oxygen saturation using random‐effect model analysis.

Five studies reported the effect of NPPV‐PSV treatment on HR in patients with CHF. A fixed‐effects model analysis was used (*p* = 0.16, *I* ^2^ = 39%), with *α* = 0.05 set as the significance level. The forest plot results demonstrated a statistically significant difference between the two groups (total effect *Z* = 16.26, MD = −10.50, 95% *CI* [−11.76, −9.23], *p* = 0.000). This suggests that NPPV‐PSV treatment is more effective in reducing HR in patients with CHF compared with conventional treatment, further reducing cardiac compliance, enhancing patient outcomes, and reducing patient mortality (see Figure 4A).

### 3.6 Respiratory Rate

The relationship between NPPV‐PSV treatment and RR was analyzed using random‐effects model analysis (*p* = 0.000, *I* ^2^ = 86%), with *α* = 0.05 set as the significance level, in five studies involving 400 patients. The forest plot results showed a statistically significant difference between the two groups (total effect *Z* = 16.50, MD = −6.44, 95% *CI* [ − 7.20, −5.67], *p* = 0.000), indicating that NPPV‐PSV treatment significantly reduced RR and improved prognosis in patients with CHF compared with conventional treatment (see Figure 4B).

### 3.7 Oxygen Saturation

We also examined the relationship between NPPV‐PSV treatment and oxygen saturation. A random‐effects model analysis was conducted (*p* = 0.000, *I* ^2^ = 84%), with *α* = 0.05 set as the significance level. The forest plot results indicated a statistically significant difference between the two groups (total effect *Z* = 12.40, MD = 0.09, 95% *CI* [0.08, 0.11], *p* = 0.000). This suggests that NPPV‐PSV treatment significantly increases oxygen saturation in patients with CHF, improves the hypoxia state of other tissues and organs, promotes tissue metabolism, and supports overall bodily functions compared with conventional treatment (see Figure 4C).

## 4 Discussion

Chronic heart failure is a syndrome caused by structural or functional cardiac abnormalities, characterized by high incidence, hospitalization, and mortality rates [18, 19]. This article evaluated the clinical efficacy and safety of NPPV‐PSV in the treatment of CHF through a systematic review and meta‐analysis. This combined method significantly enhanced the clinical effectiveness in patients with CHF, notably reducing HR and RR and improving blood oxygen saturation.

This study found that NPPV‐PSV significantly increased clinical effectiveness in patients with CHF. One study highlighted that NPPV could significantly alleviate dyspnea and enhance oxygenation, thus increasing clinical effectiveness [20]. Similarly, research on patients with acute heart failure showed that NPPV‐PSV treatment significantly reduced hospitalization and mortality rates, improving overall prognosis [21]. Furthermore, another study supported these findings, revealing that NPPV‐PSV significantly enhanced the quality of life and relieved dyspnea symptoms in patients with CHF [22]. These results demonstrate the substantial advantages of NPPV‐PSV in improving clinical effectiveness in patients with CHF. The meta‐analysis in this article also indicated that NPPV‐PSV treatment significantly reduced HR in this patient population. Studies suggest that using NPPV‐PSV can effectively reduce cardiac workload and HR, thus improving the prognosis of patients with heart failure [23, 24]. Moreover, NPPV‐PSV treatment significantly lowered the HR of patients with CHF, decreasing cardiac workload and enhancing cardiac function [25]. This effect is likely associated with improved oxygenation and reduced dyspnea. By enhancing oxygen levels and reducing carbon dioxide retention, NPPV‐PSV can lessen cardiac burden, effectively reducing HR. Additionally, the research found that NPPV‐PSV treatment significantly lowered RR of patients with CHF, improving overall prognosis [26, 27]. This meta‐analysis indicated that NPPV‐PSV treatment could significantly improve blood oxygen saturation, enhancing the overall prognosis of patients. This effect is likely linked to improved respiratory and ventilatory functions. By providing adequate ventilatory support, NPPV‐PSV can improve oxygenation status and increase blood oxygen saturation, thereby improving the overall prognosis [23]. Therefore, the curative effect profile of NPPV‐PSV is acceptable in managing CHF. However, the curative effect of this modality of treatment in acute heart failure appears to be doubtful. Until definite data is available regarding the safety of NPPV‐PSV, it would be prudent not to use it in an acute setting.

As a posteriori decision, we decided to conduct meta‐analyses of studies using disparate groups and methods. The meta‐analyses of the few studies available that provided sufficient data and reporting methods about effect sizes demonstrated that no statistical heterogeneity existed despite the presence of both clinical and methodological heterogeneity between the studies. One interpretation of these results is that the intervention effect was not influenced by factors that vary across studies (clinical heterogeneity) and was consistent across studies with respect to factors that do not vary between studies (methodological heterogeneity). Even though the study designs and methods varied, they were sufficiently homogeneous in terms of some participant characteristics, the intervention, and outcomes to provide a meaningful summary.

Despite the significant therapeutic effects of NPPV‐PSV in treating CHF as demonstrated through this systematic review and meta‐analysis, there are still some flaws and limitations in this study [28]. First, the limited number of included studies and small sample sizes might affect the reliability and generalizability of the results. Second, variations in patient selection, treatment protocols, and follow‐up durations among different studies increase the heterogeneity of the results. Third, some included studies are of low quality, which may introduce bias and affect the credibility of the outcomes. Finally, potential adverse effects of NPPV‐PSV treatment, such as airway dryness and mask discomfort, were not fully considered and discussed in this study. Future research should explore the indications and contraindications of NPPV‐PSV treatment and optimize treatment protocols to enhance efficacy and reduce adverse effects.

## 5 Conclusion

This meta‐analysis found that NPPV‐PSV treatment significantly enhanced clinical effectiveness, reduced HR and RR, and improved blood oxygen saturation. Therefore, NPPV‐PSV is an effective treatment for patients with CHF and is worthy of clinical application. However, there is no clear and unified clinical application indication for NPPV‐PSV treatment. Future research should further investigate the indications and contraindications of NPPV‐PSV treatment and conduct large‐scale, multicentre RCTs to enhance the reliability and generalizability of the results.

## Author Contributions

**Xiaohong Zhang:** conception and design of the work, statistical analysis, drafting the manuscript, critical revision of the manuscript, approval of the final manuscript. **Ye Dong:** conception and design of the work, statistical analysis, drafting the manuscript, critical revision of the manuscript, approval of the final manuscript. **Dongliang Diao:** data collection, analysis and interpretation of the data, critical revision of the manuscript, approval of the final manuscript. **Ming Li:** data collection, analysis and interpretation of the data, critical revision of the manuscript, approval of the final manuscript.

## Ethics Statement

An ethics statement was not required for this study type, no human or animal subjects or materials were used.

## Conflicts of Interest

The authors declare no conflicts of interest.

## Data Availability

All data generated or analyzed during this study are included in the article.

## References

[1] Z. Xu , H. Liu , M. Zhu , and Y. Huang , “The Inverted U‐Shaped Association Between Blood Fibrinogen and Rehospitalization Risk in Patients With Heart Failure,” Scientific Reports 14, no. 1 (July 2024): 15060, 10.1038/s41598-024-66002-3. 38956249 PMC11220044

[2] R. Eynan , R. Petrella , C. Forchuk , M. Zwarenstein , and J. Calvin , “Randomised Pilot Study Comparing a Coach to Smartphone Reminders to Aid the Management of Heart Failure (HF) Patients: Humans or Machines,” BMJ Open Quality 13, no. 3 (July 2024): e002753, 10.1136/bmjoq-2024-002753. PMC11217996 38955396

[3] Q. Chen , Z. Huang , J. Chen , et al., “Notoginsenoside R1 Attenuates Ischemic Heart Failure by Modulating MDM2/β Arrestin2‐Mediated β2‐Adrenergic Receptor Ubiquitination,” Biomedicine & Pharmacotherapy 177 (July 2024): 117004, 10.1016/j.biopha.2024.117004. 38955084

[4] Y. Terui , S. Ohura , T. Nozaki , and T. Yagi , “Pulmonary Hypertension in an Adult Patient With Congenital Central Hypoventilation Syndrome: A Case Report,” European Heart Journal—Case Reports 8, no. 3 (February 2024): ytae109, 10.1093/ehjcr/ytae109. 38454954 PMC10919382

[5] M. Vitacca and N. Ambrosino , “Non‐Invasive Ventilation as an Adjunct to Exercise Training in Chronic Ventilatory Failure: A Narrative Review,” Respiration 97, no. 1 (2019): 3–11, 10.1159/000493691. 30380534

[6] M. Peers de Nieuwburgh , C. Cecarelli , D. Weinberg , K. C. Yang , H. M. Herrick , and E. E. Foglia , “Outcomes After Delivery Room Positive Pressure Ventilation in Late Preterm and Term Infants,” Resuscitation Plus 19 (May 2024): 100670, 10.1016/j.resplu.2024.100670. 38881597 PMC11177047

[7] H. Jiang , Y. Han , C. Xu , J. Pu , and B. He , “Noninvasive Positive Pressure Ventilation in Chronic Heart Failure,” Canadian Respiratory Journal 2016 (2016): 3915237, 10.1155/2016/3915237. 27891061 PMC5116333

[8] J. Masip , “Noninvasive Ventilation in Acute Heart Failure,” Current Heart Failure Reports 16, no. 4 (2019): 89–97, 10.1007/s11897-019-00429-y. 31147960

[9] J. F. Masa , B. Mokhlesi , I. Benítez , et al., “Long‐Term Clinical Effectiveness of Continuous Positive Airway Pressure Therapy Versus Non‐Invasive Ventilation Therapy in Patients With Obesity Hypoventilation Syndrome: A Multicentre, Open‐Label, Randomised Controlled Trial,” Lancet 393, no. 10182 (April 2019): 1721–1732, 10.1016/S0140-6736(18)32978-7. 30935737

[10] A. Valipour , W. Cozzarini , and O. C. Burghuber , “Non‐Invasive Pressure Support Ventilation in Patients With Respiratory Failure Due to Severe Acute Cardiogenic Pulmonary Edema,” Respiration 71, no. 2 (March/April 2004): 144–151, 10.1159/000076675. 15031569

[11] J. Masip , W. F. Peacock , S. Price , et al., “Indications and Practical Approach to Non‐Invasive Ventilation in Acute Heart Failure,” European Heart Journal 39, no. 1 (Jnuary 2018): 17–25, 10.1093/eurheartj/ehx580. 29186485 PMC6251669

[12] P. B. Murphy , C. Davidson , M. D. Hind , et al., “Volume Targeted Versus Pressure Support Non‐Invasive Ventilation in Patients With Super Obesity and Chronic Respiratory Failure: A Randomised Controlled Trial,” Thorax 67, no. 8 (August 2012): 727–734, 10.1136/thoraxjnl-2011-201081. 22382596

[13] F. M. Struik , R. T. M. Sprooten , H. A. M. Kerstjens , et al., “Nocturnal Non‐Invasive Ventilation in Copd Patients With Prolonged Hypercapnia after Ventilatory Support for Acute Respiratory Failure: A Randomised, Controlled, Parallel‐Group Study,” Thorax 69, no. 9 (September 2014): 826–834, 10.1136/thoraxjnl-2014-205126. 24781217

[14] M. S. Stefan , B. H. Nathanson , T. L. Higgins , et al., “Comparative Effectiveness of Noninvasive and Invasive Ventilation in Critically Ill Patients With Acute Exacerbation of Chronic Obstructive Pulmonary Disease,” Critical Care Medicine 43, no. 7 (July 2015): 1386–1394, 10.1097/CCM.0000000000000945. 25768682 PMC4470719

[15] T. Köhnlein , W. Windisch , D. Köhler , et al., “Non‐Invasive Positive Pressure Ventilation for the Treatment of Severe Stable Chronic Obstructive Pulmonary Disease: A Prospective, Multicentre, Randomised, Controlled Clinical Trial,” Lancet Respiratory Medicine 2, no. 9 (September 2014): 698–705, 10.1016/S2213-2600(14)70153-5. 25066329

[16] E. Márquez‐Martín , F. O. Ruiz , P. C. Ramos , J. L. López‐Campos , B. V. Azcona , and E. B. Cortés , “Randomized Trial of Non‐Invasive Ventilation Combined With Exercise Training in Patients With Chronic Hypercapnic Failure Due to Chronic Obstructive Pulmonary Disease,” Respiratory Medicine 108, no. 12 (December 2014): 1741–1751, 10.1016/j.rmed.2014.10.005. 25456710

[17] C. N. Di Leone , C. P. Diniz , T. M. Vieira de Araújo , et al., “Aerobic Exercise Simultaneous With Non‐Invasive Ventilation Reduces the Length of Stay in Intensive Care in Patients With Heart Failure: A Randomised Clinical Trial,” European Journal of Physiotherapy 26, no. 3 (2023): 176–184, 10.1080/21679169.2023.2229392.

[18] J. Shrestha and S. Done , “An Overview of Chronic Heart Failure,” Nursing Standard 38, no. 8 (2023): 43–49, 10.7748/ns.2023.e12049. 37394965

[19] J. L. Kelly , J. Jaye , R. E. Pickersgill , M. Chatwin , M. J. Morrell , and A. K. Simonds , “Randomized Trial of ‘Intelligent’ Autotitrating Ventilation Versus Standard Pressure Support Non‐Invasive Ventilation: Impact on Adherence and Physiological Outcomes,” Respirology 19, no. 4 (May 2014): 596–603, 10.1111/resp.12269. 24661390

[20] F. Zhang , G. Zhou , L. Guo , F. Lu , and G. Zhou , “Comparison of Clinical Efficacy of Metoprolol Combined With Irbesartan and Hydrochlorothiazide and Non‐Invasive Ventilator in the Emergency Treatment of Patients With Severe Heart Failure,” Experimental and Therapeutic Medicine 16, no. 6 (2018): 5059–5066, 10.3892/etm.2018.6828. 30542460 PMC6257578

[21] V. Comellini , A. M. G. Pacilli , and S. Nava , “Benefits of Non‐Invasive Ventilation in Acute Hypercapnic Respiratory Failure,” Respirology 24, no. 4 (2019): 308–317, 10.1111/resp.13469. 30636373

[22] M. Sakuraya , H. Okano , T. Masuyama , S. Kimata , and S. Hokari , “Efficacy of Non‐Invasive and Invasive Respiratory Management Strategies in Adult Patients With Acute Hypoxaemic Respiratory Failure: A Systematic Review and Network Meta‐Analysis,” Critical Care 25, no. 1 (November 2021): 414, 10.1186/s13054-021-03835-8. 34844655 PMC8628281

[23] D. L. Grieco , S. M. Maggiore , O. Roca , et al., “Non‐Invasive Ventilatory Support and High‐Flow Nasal Oxygen as First‐Line Treatment of Acute Hypoxemic Respiratory Failure and ARDS,” Intensive Care Medicine 47, no. 8 (August 2021): 851–866, 10.1007/s00134-021-06459-2. 34232336 PMC8261815

[24] K. Hussein , “Non Invasive Spontaneous Dual Ventilation in Critically Ill Patients With Chronic Obstructive Pulmonary Disease,” Egyptian Journal of Chest Diseases and Tuberculosis 65, no. 1 (2016): 99–104, 10.1016/j.ejcdt.2015.08.016.

[25] M. L. Duiverman , J. M. Vonk , G. Bladder , et al., “Home Initiation of Chronic Non‐Invasive Ventilation in COPD Patients With Chronic Hypercapnic Respiratory Failure: A Randomised Controlled Trial,” Thorax 75, no. 3 (March 2020): 244–252, 10.1136/thoraxjnl-2019-213303. 31484786 PMC7063397

[26] C. R. Osadnik , V. S. Tee , K. V. Carson‐Chahhoud , J. Picot , J. A. Wedzicha , and B. J. Smith , “Non‐Invasive Ventilation for the Management of Acute Hypercapnic Respiratory Failure Due to Exacerbation of Chronic Obstructive Pulmonary Disease,” Cochrane Database of Systematic Reviews 2017, no. 7 (July 2017): CD004104, 10.1002/14651858.CD004104.pub4. PMC6483555 28702957

[27] G. E. Carpagnano , R. Sabato , D. Lacedonia , et al., “New Non Invasive Ventilator Strategy Applied to COPD Patients in Acute Ventilator Failure,” Pulmonary Pharmacology & Therapeutics 46 (October 2017): 64–68, 10.1016/j.pupt.2017.08.009. 28823948

[28] E. R. Araújo , I. D. Bezerra Nogueira , P. E. E Silva Barbosa , and P. A. M. Silva Nogueira , “Effects of Non‐Invasive Ventilation With Different Modalities in Patients Undergoing Heart Surgery: Protocol for a Randomized Controlled Clinical Trial,” PLoS One 19, no. 6 (June 2024): e0304569, 10.1371/journal.pone.0304569. 38889140 PMC11185470

